# Mediterranean diet, gut microbiota and health: when age and calories do not add up!

**DOI:** 10.1136/gutjnl-2020-320781

**Published:** 2020-03-13

**Authors:** Patrice D Cani, Matthias Van Hul

**Affiliations:** UCLouvain, Universite catholique de Louvain, WELBIO- Walloon Excellence in Life Sciences and BIOtechnology, Louvain Drug Research Institute, Metabolism and Nutrition Research Group, Brussels, Belgium

**Keywords:** nutrition, obesity, diabetes mellitus, colonic microflora, intestinal microbiology

A healthy diet is generally recognised as a diet that supports the physiological and energetic requirements of the body and provides sufficient, though not excessive, amounts of micronutrients and macronutrients. Despite this self-evident definition, the implementation of this basic principle has proven very difficult in modern western society. The benefits of adopting a Mediterranean diet (MedDiet) were already scientifically described more than 50 years ago,^1^ when a reduction in cardiovascular disease risk was observed among populations whose nutritional habits were consistent with those of people from the Mediterranean basin.^2^ Today, adherence to a MedDiet has been associated with lower mortality, reductions in obesity, type 2 diabetes, low-grade inflammation, cancer, Alzheimer’s disease and depression, and, more recently, the delayed onset of Crohn’s disease.^3–5^

Surprisingly, the exact mechanisms of action are not yet fully understood, and various hypotheses have been proposed to explain the potential beneficial effects of the MedDiet. Among them, a presumed link between the MedDiet and the gut microbiota was put forward not more than a few years ago; this idea is therefore considered relatively new.

In *GUT*, two studies conducted in humans have now made progress towards a better understanding of the role of the gut microbiota and the MedDiet in disease risk factors.^6 7^

In the first paper, Meslier and colleagues studied healthy overweight or obese subjects with sedentary lifestyles who habitually consumed low amounts of fruits and vegetables during an 8-week follow-up.^6^ In this randomised controlled study, 43 subjects were assigned to the MedDiet group, in which they received a personalised diet adapted to their habitual energy intake. The remaining 39 subjects continued on their regular diet. Among the different food items proposed to the MedDiet group were fewer meat and refined-cereal products and more fish, fruits, vegetables, legumes and whole grains, as well as a daily serving of nuts. Thus, this diet doubled the total amount of fibre, increased the ratio of vegetable to animal protein by 2.5-fold and included fewer saturated fatty acids and more polyunsaturated fatty acids.

One original aspect of this protocol is that since the MedDiet matched their habitual energy intake, the subjects were instructed not to change their caloric intake but rather the quality of their diet. The provided calories were calculated based on self-reported food diaries, and caution about the accuracy of this method is therefore warranted. However, this is a common limitation within the field due to the lack of a reasonable alternative. A second striking finding is that the MedDiet was accompanied by a reduction in total plasma cholesterol only 4 weeks after the beginning of the diet, followed by a lower total, high-density lipoprotein (HDL), low-density lipoprotein (LDL) cholesterol and faecal bile acids at the end of the intervention. Metabolomic analyses of the faeces, urine and blood revealed a clear shift after the implementation of the MedDiet, and this shift was characterised by significant changes in different metabolomic biomarkers (eg, higher urolithins, tryptophan betaine and oxindole-3-acetic acid and lower carnitine, p-cresol and indoxyl sulfate) considered putative signatures of adherence to the MedDiet. In addition to the metabolome, the authors found specific changes in the gut microbiota composition, such as an increased abundance of *Faecalibacterium prausnitzii* and *Roseburia* and a lower abundance of *Ruminococcus gnavus* and *R. torques*. Interestingly, the variation in insulin resistance was linked to specific bacteria, and the subjects who reduced their index of insulin resistance had higher baseline levels of *Bacteroides uniformis* and *B. vulgatus* and lower levels of *Prevotella copri*. As previously observed by Dao *et al*, both baseline microbiota and microbial richness were inversely associated with hs-C-reactive protein and insulin resistance.^8^

Surprisingly, the marked increase in dietary fibre intake resulted in a higher abundance of butyrate-producing bacteria and in higher levels of butyrate genes, yet the authors did not find any changes in the amount of faecal short-chain fatty acids (SCFAs). This strongly brings into question whether measuring faecal SCFAs is an adequate biomarker.

The second interesting paper published in *GUT* investigates the effect of a MedDiet followed for 1 year in 612 elderly non-frail and prefrail subjects across five countries.^6^ Ageing has been associated with changes in the gut microbiota composition and higher inflammation levels. This process is accelerated by a diet with a low diversity of food components.^9^ In this study, the authors found that adherence to MedDiet led to a higher abundance of different taxa that are positively associated with markers of lower frailty and better cognitive function but also negatively correlated with markers of inflammation. Interestingly, these observations were independent of body mass index and age. This strongly suggests that the MedDiet intervention drove the changes in the gut microbiota composition. According to the authors, this was mainly due to the ingestion of fibre, certain vitamins (C, B6, B9) and various minerals. Conversely, in the control group, the changes were mostly linked to a higher increase in total fat intake. Although at baseline, there were already some differences in the gut microbiota composition among countries (mainly linked to local dietary habits), the diversity was similar and adherence to the MedDiet was associated with an attenuated loss of microbiome diversity. Seventy-five operational taxonomic unit (OTUs) provided a high predictive performance to identify the microbiome response to the MedDiet. Additionally, 44 OTUs displayed a positive association with adherence to the diet, that is, had a higher abundance when the MedDiet was strictly observed, whereas 45 OTUs were negatively associated with adherence to the diet. The authors called these OTUs either ‘diet positive’ or ‘diet negative’. The diet-positive OTUs included *F. prausnitzii*, *Eubacterium rectale*, *Roseburia*, *Bacteroides thetaiotaomicron, P. copri* and *Anaerostipes hadrus*. The diet-negative OTUs included *R. torques*, *Collinsella aerofaciens*, *Coprococcus comes*, *Dorea formicigenerans*, *Clostridium ramosum*, *Veillonella dispar*, *Flavonifractor plautii* and *Actinomyces lingnae*. It is important to note that these different taxa were shared among countries, reinforcing the fact that despite different baselines and specific dietary habits across countries, the MedDiet drives the gut microbiota composition in a consistent manner.

This study did not include metabolomic analyses, and the authors decided to infer the faecal microbial metabolites based on the metagenomic analysis. They speculated that the MedDiet promotes SCFA production while decreasing bile acids and cresols. However, predicting the functional metabolic profiles of the gut microbiome by using the corresponding species is risky and is likely the major weakness of this study. Having said that, the authors confirmed their assumptions about the bile acid profile by measuring the different plasma bile acids, which were consistent with the postulated microbial profile.

On the other hand, one may even argue that having both metagenomic and metabolomic analyses in faeces still only provides part of the complex story behind diet–host interactions. For example, in the first study discussed above,^6^ a profound change in the diet (ie, higher levels of fibre) led to a higher abundance of classical SCFA producers. Curiously, although higher levels of SCFAs were expected, an actual measurement in the faeces revealed that the SCFA levels remained unaffected by the diet. This illustrates the limitations of studying the microbiota composition and their metabolites in faecal matter and the risk of extrapolating the overall effects of specific taxa or metabolites on physiological effects when limited time points and biological compartments are available. Another marked difference between the two studies is the designation of *P. copri* as either a ‘positive’ or a ‘negative’ bacteria. As previously discussed,^10^ the role of *P. copri* is again presented as ambiguous. Indeed, although both MedDiet studies reached the same general conclusions, the presence of *P. copri* as a negative or positive OTU is a key example of how misleading a mere association can be in the study of the gut microbiota.

In conclusion, these two outstanding studies support the fact that (1) it is not the quantity of calories per se that matters but the quality of the diet and (2) even in subjects of advanced age, adherence to a MedDiet is rapidly associated with different metabolic effects and reduced disease risk factors. Nevertheless, these studies also underscore the challenge of precisely dissecting the mechanisms by which a MedDiet can affect human health. Indeed, although the vast majority of the results are consistent between studies, some potential markers (ie, taxa and metabolites) may appear discrepant and even contrasting, thereby supporting the need for appropriate proof-of-concept studies to ascertain the role of one or several specific bacteria as potential next-generation beneficial bacteria to provide protection against specific diseases.
